# Supplementary material for: Kuura—An automated workflow for analyzing WES and WGS data
Source: PLoS One. 2024 Jan 18;19(1):e0296785. doi: 10.1371/journal.pone.0296785 (PMC10796025; doi:10.1371/journal.pone.0296785)
Supplement: S1 File — (DOCX) [file pone.0296785.s001.docx]

**Kuura - An automated workflow for analyzing WES and WGS data**

Kuura is an open-source application for automated sequencing data analysis. The pipeline is written in nextflow, and the running environment is provided in a docker container to make them scalable and reproducible. This supplementary file provides information on how to install *docker*, *git*, *nextflow* and how to run Kuura pipeline for DNA-seq data analysis.

**Installation:**

**Step 1: Installing *docker*:**

Installing *docker* on Ubuntu Linux systems requires versions 18.04 or higher.

Setting up *docker* repository:

1. Update advanced package tool (apt) index and install packages to allow it to use a repository over HTTPS.

| # sudo apt-get update # sudo apt-get install \  ca-certificates \  curl \  gnupg \  lsb-release |
| --- |

1. Add *docker*’s official GPG key:

| # sudo mkdir -p /etc/apt/keyrings # curl -fsSL https://download.docker.com/linux/ubuntu/gpg \| sudo gpg --dearmor -o /etc/apt/keyrings/docker.gpg |
| --- |

1. Set up the repository:

| # echo \ "deb [arch=$(dpkg --print-architecture) signed-by=/etc/apt/keyrings/docker.gpg] https://download.docker.com/linux/ubuntu \  $(lsb_release -cs) stable" \| sudo tee /etc/apt/sources.list.d/docker.list > /dev/null |
| --- |

1. Install *docker* engine:

| # sudo apt-get update # sudo apt-get install docker-ce docker-ce-cli containerd.io docker-compose-plugin |
| --- |

1. Verify *docker* is installed correctly:

| # sudo service docker start # sudo docker run hello-world |
| --- |

If you prefer to install a specific version of docker you can find more information on the *docker* website (<https://docs.docker.com/engine/install/ubuntu/>).

**Step 2: Download pipeline from github:**

The source code of the pipeline can be downloaded from the github repository.

| # git clone <https://github.com/dhanaprakashj/kuura_pipeline> |
| --- |

**Step 3: Setting up *docker* images (need root permissions):**

The pipeline uses official *docker* images for tools which are made available by the developers (*GATK*, *mosdepth*, *deepvariant* & *ensembl-vep*) and for the remaining tools a custom docker image has been developed.

- Pull images from DockerHub

| # docker pull broadinstitute/gatk:4.3.0.0 # docker pull quay.io/biocontainers/mosdepth:0.2.4--he527e40_0 # docker pull google/deepvariant:1.4.0 # docker pull ensemblorg/ensembl-vep:release_107.0 |
| --- |

- Build custom *docker* image.

| # cd kuura_pipeline/docker/ # docker build -t dna-seq-pipeline:0.1 . |
| --- |

- Check the images and tags.

| # docker image ls |
| --- |

**Step 3: Installing *nextflow*:**

Change into your preferred directory and install *nextflow* executable.

| # cd <nextflow_directory> # wget -qO- https://get.nextflow.io \| bash # chmod +x nextflow # export PATH="<nextflow directory>:$PATH" # nextflow -version |
| --- |

**Running the DNA-Seq pipeline:**

**Step 1:** Download and set up reference genome and required annotation files.

**Step 2:** Download sample datasets. The publicly available datasets used in the evaluation were downloaded from the EBI FTP server:

HG001:
Exome sequencing of Homo sapiens: HG001 with Illumina NovaSeq 6000 IDT capture - SRR14724473
<ftp://ftp.sra.ebi.ac.uk/vol1/fastq/SRR147/073/SRR14724473/SRR14724473_1.fastq.gz>

<ftp://ftp.sra.ebi.ac.uk/vol1/fastq/SRR147/073/SRR14724473/SRR14724473_2.fastq.gz>

HG002:
Exome sequencing of Homo sapiens: HG002 with Illumina NovaSeq 6000 IDT capture - SRR14724472
<ftp://ftp.sra.ebi.ac.uk/vol1/fastq/SRR147/072/SRR14724472/SRR14724472_1.fastq.gz>

<ftp://ftp.sra.ebi.ac.uk/vol1/fastq/SRR147/072/SRR14724472/SRR14724472_2.fastq.gz>

HG005:
Exome sequencing of Homo sapiens: HG005 with Illumina NovaSeq 6000 IDT capture - SRR14724469
<ftp://ftp.sra.ebi.ac.uk/vol1/fastq/SRR147/069/SRR14724469/SRR14724469_1.fastq.gz>

<ftp://ftp.sra.ebi.ac.uk/vol1/fastq/SRR147/069/SRR14724469/SRR14724469_2.fastq.gz>

The IDT capture bed file used for coverage analysis and hap.py validation was obtained from:

https://storage.googleapis.com/deepvariant/exome-case-study-testdata/idt_capture_novogene.grch38.bed

**Step 3: Get to know the pipeline setup:**

The files in the dnaseq repository are listed below.

- bin/main.nf
- nextflow.config
- configuration/
  - execution-parameters.config
  - default.config
  - testing.config

3.1: bin/main.nf:

This is the main script file describing the sequence of the analysis to be done and defines the parameters for the run. Any addition/removal of analysis steps are done in this file. An example process is given below,

| process QualityAssessment {   label "utuprcagenetics"   maxForks params.lightResourceForkThreshold   tag "FASTQC on $fastq"   publishDir "$params.outputDir/FASTQC"   input:  file(fastq) from allFastq_ch   output:  file("${outputZIP}")  file("${outputHTML}")  val("DoneInitialFastQC") into doneFastQC    script:  outputZIP = "${fastq}".replaceFirst(/.fastq.gz$/, "_fastqc.zip")  outputHTML = "${fastq}".replaceFirst(/.fastq.gz$/, "_fastqc.html")   """  fastqc -q -o . ${fastq}  """ } |
| --- |

In the above example, the process is named QualityAssessment. The parameters defined inside the process are as follows:

1. label - The label directive allows the user to annotate a process with a specific keyword. In this pipeline we make use of the label directive with ‘withLabel’ process selector (step 3.3) to define docker images to be used for the specific process.
2. maxForks - Maximum number of process instances that can be executed in parallel. The parameters ‘params.highResourceForkThreshold’ and ‘params.lightResourceForkThreshold’ can be used to adjust the number of forks for a process depending on the computational resources available. There are other directives such as ‘maxErrors’, ‘maxRetries’ and memory that can be used to modify the resources made available for running the pipeline.
3. tag - tag directive enables association of each process execution with custom label. This label makes it easier to identify processes in log file and execution summaries.
4. publishDir - This directive allows defining the output folder for that specific process.
5. input - Input block defines from which channels the process expects to receive data. There can be more than one input for a process but not more than one input block. The basic structure of the input block is,

| <input qualifier> <input name> [from <source channel>] [attributes] |
| --- |

- input qualifier - refers to the types of input that is expected.
- input name - refers to the name of the expected input.
- from - keyword to specify source of input.
- source channel - channel from which input is expected.
- attributes - optional attributes to select input can be specified using this directive.

More information on the input block can be found from -

<https://www.nextflow.io/docs/latest/process.html#inputs>

1. output - Output block defines the channels that carry data from the process once it is complete. There can be more than one output for a process, and they can be defined together in one output block. The basic structure of the output block is as follows:

| <output qualifier> <output name> [into <target channel>[,channel,..]] [attribute [,..]]   - output qualifier - refers to the types of output that is expected. - output name - refers to the name of the expected output. - into - keyword to specify output to channel. - target channel - channel over which output is sent to next process. - attributes - optional conditional attributes for specifying outputs to channels. |
| --- |

More information on the output block can be found from -

<https://www.nextflow.io/docs/latest/process.html#outputs>

1. script - Script block defines the command that should be executed by the process to carry out its task. More information on scripts can be found from -

<https://www.nextflow.io/docs/latest/process.html#script>

1. Shell (execution) block - The shell block is denoted by triple quotes and the shell commands to be executed by the process are defined here.

In-depth information on defining a process can be obtained from -

[https://www.nextflow.io/docs/latest/process.html#](https://www.nextflow.io/docs/latest/process.html)

3.2 nextlfow.config:

The nextflow.config file contains scripts to include custom config profiles for running the analysis. A custom config file can be defined as follows,

| Profiles {  custom {  includeConfig 'configuration/custom.config'  includeConfig 'configuration/execution-parameters.config'  } } |
| --- |

Profiles can also be defined to include information such as the process executor, memory and so on. More information on creating custom profiles and their scopes is available from *nextflow* documentation ([https://www.nextflow.io/docs/latest/config.html?highlight=params#](https://www.nextflow.io/docs/latest/config.html?highlight=params)).

3.3 configuration/execution-parameters.config:

The execution-parameters.config file contains information on the containers and the containerization technology (*docker*) that is used for running the pipeline. The file is divided into two blocks - *docker* configurations and *docker* profiles for specific processes.

The parameters for the *docker* container can be defined in the first block, more information on docker scope can be found from <https://www.nextflow.io/docs/latest/config.html#config-docker>.

| docker {  enabled = true  temp = 'auto'  runOptions = "--volume /mount/:/mount/ -u \$(id -u):\$(id -g)"  } |
| --- |

The second block contains process selectors for defining *docker* containers to be used with processes tagged with specific labels.

| process {  withLabel: 'utuprcagenetics' {  container = 'dna-seq-pipeline:0.1'  } } |
| --- |

3.4 configuration/default.config & configuration/testing.config:

This is the configuration profile where the parameters for running the pipeline are defined. The parameters are listed in step 4.

**Step 4:** Configure the nextflow parameters,

| **Parameters** | **Description** |
| --- | --- |
| params.outputDir | Full path to the directory where the soft links of the files are stored. |
| params.groupIdentifier | Unique identifiers for your sample. |
| params.libraryType | Specify whether the sample is WES/WGS. |
| params.seqPlatform | Specify sequencing platform. |
| params.noAdaptor | If set to TRUE, uses fastp for adapter trimming, if set to FALSE uses cutadapt and adapters mentioned in params.adaptors |
| params.exomeRegionsBed | Exome targeted regions in the form of a bed file. |
| params.dataDir | Full path to the directory where the input files are stored. |
| params.allFastq | Full path to the fastq files identifying them with a glob pattern. |
| params.reads | Full path to the fastq files with glob pattern to identify matching reads. |
| params.adaptors | Adapter information present in the sample. |
| params.alignmentReferenceDir | Full path to the directory where the FASTA reference file is stored. |
| params.alignmentReference | Full path to the FASTA reference file. |
| params.dbSNPcommon | dbSNP filter of common variants. |
| params.millsReference | Databases of known polymorphic sites used to exclude regions around known polymorphisms from analysis |
| params.dbSNPReference | dbSNP database (Human variations without clinical assertions). |
| params.hapmapReference | HapMap genotypes and sites. |
| params.omniReference | omni_2.5 genotypes for 1000 genomes samples and sites. |
| params.reference1000G | 1000 genomes project high confidence snps. |
| params.knownIndels | Broad Institute list of know indels vcf. |
| params.heavyResourceForkThreshold | Maximum number of process instances that can be executed in parallel. |
| params.lightResourceForkThreshold | Maximum number of process instances that can be executed in parallel. |

**Step 5:** Run the pipeline:

After defining all the parameters, the pipeline can be run from the dnaseq folder by issuing the following command,

| nextflow run bin/main.nf -work-dir <workspace directory> -profile <profileName> |
| --- |

Nextflow will create the workflow directory and place all the intermediate result files there and their symbolic links in the respective output folder.

For more information on using the pipeline, kindly refer to the git repository.
